# Exploring the Molecular Mechanisms of Psoriasis and Other Skin Disorders: Unveiling New Insights

**DOI:** 10.3390/ijms26094061

**Published:** 2025-04-25

**Authors:** Elisabetta Palazzo

**Affiliations:** DermoLAB, Department of Surgical, Medical, Dental and Morphological Science, University of Modena and Reggio Emilia, via Del Pozzo 71, 41124 Modena, Italy; elisabetta.palazzo@unimore.it

Recent research into psoriasis and other skin diseases has made remarkable strides in elucidating the molecular mechanisms that drive inflammation and skin pathology. The growing body of evidence is reshaping our understanding of these conditions and revealing novel therapeutic targets that could improve patient outcomes. This Special Issue, “Molecular and Cellular Mechanisms of Skin Diseases”, presents novel research concerning biomarker identification, molecular profiling, and potential treatments.

Bozò et al. explore the expression of cytokines and chemokines in psoriatic non-lesional (PS-NL) and resolved skin from healthy skin. This research indicates that mild and severe stages of psoriasis can be distinguished by the levels of these inflammatory molecules, even when visible lesions are absent. These findings emphasize the potential of non-lesional skin as critical to understanding the progression of psoriasis and as a marker for early intervention. The skin environment, characterized by the altered expression of immune molecules, plays a crucial role in initiating and sustaining disease activity, even in regions unaffected by lesions. Analyzing the cytokine and chemokine profiles in these areas may provide new insights into inflammation stages, suggesting possible biomarkers that can inform treatment decisions [1].

Similarly, Vitamin D has been shown to have a role in psoriasis [2]. Zanghaneh et al. examined the expression of the Vitamin D receptor (VDR) on peripheral blood mononuclear cells (PBMCs) in both patients with psoriasis and healthy individuals and the effect of Etanercept on Vitamin D expression. The results indicate that Vitamin D influences immune modulation, increasing the expression of CD14+ monocytes; however, this mechanism remains a complex area of study in psoriasis. Overall, this could be crucial for understanding how Vitamin D might be utilized for therapeutic purposes. By exploring the connection between Vitamin D signaling and immune cell activity, researchers aim to clarify the ongoing debates surrounding Vitamin D supplementation in psoriasis management [3].

Considering the “interleukin profiling” of skin disorders, the study by Wiegmann et al. explores the role of interleukins (IL-4, IL-13, and IL-31) and their receptors in atopic dermatitis (AD) and chronic nodular prurigo (CNPG). The results showed a high expression of IL-4 and IL-13 and the receptors IL-13RA1 and IL-13RA2 in skin samples from patients with AD compared to healthy controls, suggesting the central role of these mediators in inflammation and itching. In contrast, CNPG showed a low expression of IL-13RA2, indicating a dysfunction in regulatory mechanisms, which may contribute to persistent chronic itching. Correlations with itch intensity, measured by the VAS scale, showed a link between IL-13RA1 expression and itch severity in AD. Boruta algorithm-based analysis identified IL-4, IL-13, IL-4R, IL-13RA1, and IL-13RA2 as crucial parameters for distinguishing AD and CNPG from healthy controls. These findings offer new insights for targeted therapies aimed at treating chronic itch in these conditions [4].

Via proteomic analysis, Kromann et al. discovered a significant overexpression of complement cascade proteins in the plasma of psoriasis patients, alluding to the fact that the complement system may play a key role in disease onset and progression [5]. This finding suggests that the complement pathway could serve as a biomarker for disease severity and as a potential therapeutic target. The study provides insights into the inflammatory nature of psoriasis, highlighting the importance of immune system dysregulation.

Beyond these findings, research into the genetic and epigenetic factors affecting psoriasis has furthered our understanding of how environmental factors, such as seasonal changes, can influence disease activity [6]. A novel comprehensive review on psoriasis seasonality emphasizes the interplay between genetics, epigenetics, and environmental exposures, such as UV radiation and pollution. This interplay could explain the disease’s flare-ups during certain times of the year, shedding light on how environmental triggers interact with genetic predispositions to exacerbate the disease [7].

In addition to exploring the causes of psoriasis, attention is also being directed toward innovative treatments. For example, a study by Li and colleagues on the therapeutic effects of a combination of ingredients, including Lecigel and Cetiol^®^CC, demonstrated significant promise in treating psoriasis-like skin conditions in mice. Their results indicate that this treatment improves imiquimod-induced skin phenotypes by reducing serum levels of inflammatory cytokines and the expression of several factors associated with angiogenesis and proliferation/differentiation in keratinocytes, such as TGFβ. This suggests the potential for new topical treatments that could reduce inflammation and promote healing in psoriatic lesions [8].

Finally, Arakawa and collaborators show how UVB phototherapy may suppress the psoriatic autoimmune response against melanocytes by downregulating HLA-C expression [9]. This study provides compelling evidence that the downregulation of HLA-C expression on melanocytes induced by UVB phototherapy is essential to the therapeutic efficacy of this psoriasis treatment. The findings demonstrate that UVB irradiation reduces the immunogenicity of melanocytes by suppressing key IFN-γ-induced transcriptional regulators. Additionally, UVB increases the expression of miR-148a, which specifically targets and inhibits HLA-C expression. These results highlight that UVB’s targeted effect on IFN-γ-induced antigen presentation pathways is a crucial mechanism underlying its selective effectiveness in T-cell-mediated skin diseases such as psoriasis, suggesting potential new targets for improving psoriasis treatment.

In the context of skin diseases characterized by the dysregulation of proliferation/differentiation balance, the transcription factor ΔNp63 plays a pivotal role in maintaining the integrity of stratified epithelial tissues by regulating the expression of distinct target genes involved in lineage specification, cell stemness, cell proliferation, and differentiation. La Banca et al. identified the ABC transporter subfamily member *ABCC1* as a novel ΔNp63 target gene in immortalized human keratinocytes and in squamous cell carcinoma (SCC) cells, which correlates with a keratinocyte differentiation program [10]. Humanized *hABCC1* knock-out mice did not exhibit epithelial alternation but showed a marked reduction in the inflammation-mediated proliferation of keratinocytes, revealing that ABCC1 might be involved in the regulation of keratinocyte proliferation in response to inflammatory/proliferative signals, which is common in squamous cell carcinomas.

Researchers are also exploring how immune mechanisms contribute to other skin diseases, such as pemphigus vulgaris, an autoimmune disorder where IgG is produced against desmoglein 1 and 3 [11]. Consequently, this disrupts the adhesion between skin cells, leading to painful blisters and erosions. Pemphigus vulgaris-IgG binding involves p38MAPK-signaling-dependent caspase-3 activation. By targeting caspase-3, a key protein involved in cell detachment, Pacheco-Tovar and coworkers have demonstrated that inhibiting this pathway can significantly reduce the skin damage induced by pemphigus vulgaris autoantibodies. This leads to the possibility of developing potential therapies that could prevent severe skin detachment, which is characteristic of the disease.

Finally, the study by Bchetnia et al. [12] explores the molecular and cellular mechanisms underlying epidermolysis bullosa simplex (EBS), a rare skin disorder caused primarily by mutations in the KRT5 and KRT14 genes. It discusses how keratinocyte fragility leads to blister formation and highlights key pathways such as oxidative stress, inflammation, and autophagy. This review also covers potential therapies, including gene editing, stem cell therapy, and small molecules targeting these mechanisms. Overall, it provides a comprehensive overview of current research and future treatment prospects.

The studies collected in this Special Issue enhance our knowledge of skin diseases and signal a future where more personalized and effective therapies may become available. By combining advanced molecular research with clinical insights, the medical community is becoming increasingly equipped to address the complexities of skin diseases, offering hope for improved management and patient outcomes.

In conclusion, the molecular exploration of skin diseases such as psoriasis reveals novel insights that promise to revolutionize treatment strategies. From uncovering immune pathways to identifying biomarkers and testing innovative therapies, these studies underscore the critical need for continued research. As we deepen our understanding of the mechanisms underlying skin diseases, we move closer to developing more effective, targeted interventions that could dramatically improve the lives of people affected by these conditions.

## References

[1] Bozó R., Flink L.B., Ambrus B., Ghaffarinia A., Koncz B., Kui R., Gyulai R., Kemeny L., Bata-Csorgo Z. (2024). The Expression of Cytokines and Chemokines Potentially Distinguishes Mild and Severe Psoriatic Non-Lesional and Resolved Skin from Healthy Skin and Indicates Different Stages of Inflammation. Int. J. Mol. Sci..

[2] Barrea L., Savanelli M.C., Di Somma C., Napolitano M., Megna M., Colao A., Savastano S. (2017). Vitamin D and its role in psoriasis: An overview of the dermatologist and nutritionist. Rev. Endocr. Metab. Disord..

[3] Zanghaneh A.J., Elmelid A., Gillstedt M., Ahmic O., Andersson B., Osmancevic A. (2024). The Expression of Vitamin D Receptor on Peripheral Blood Mononuclear Cells in Patients with Psoriasis. Int. J. Mol. Sci..

[4] Wiegmann H., Renkhold L., Zeidler C., Agelopoulos K., Ständer S. (2024). Interleukin Profiling in Atopic Dermatitis and Chronic Nodular Prurigo. Int. J. Mol. Sci..

[5] Kromann B., Niu L., Møller L.B.P., Sølberg J., Sulek K., Gyldenløve M., Dyring-Andersen B., Skov L., Løvendorf M.B. (2024). Unbiased Proteomic Exploration Suggests Overexpression of Complement Cascade Proteins in Plasma from Patients with Psoriasis Compared with Healthy Individuals. Int. J. Mol. Sci..

[6] Jensen K.K., Serup J., Alsing K.K. (2022). Psoriasis and seasonal variation: A systematic review on reports from Northern and Central Europe-Little overall variation but distinctive subsets with improvement in summer or wintertime. Skin Res. Technol..

[7] Niedźwiedź M., Skibińska M., Ciążyńska M., Noweta M., Czerwińska A., Krzyścin J., Narbutt J., Lesiak A. (2024). Psoriasis and Seasonality: Exploring the Genetic and Epigenetic Interactions. Int. J. Mol. Sci..

[8] Li C.C., Lin C.C., Lee C.Y., Sheu M.L., Tsai Y.C., Tsai C.Y., Wu H.-T., Wu R.-J., Lai D.-W. (2024). Therapeutic Effect of Lecigel, Cetiol®CC, Activonol-6, Activonol-M, 1,3-Propanediol, Soline, and Fucocert® (LCAA-PSF) Treatment on Imiquimod-Induced Psoriasis-like Skin in Mice. Int. J. Mol. Sci..

[9] Arakawa Y., Arakawa A., Vural S., He M., Vollmer S., Prinz J.C. (2025). Down-Regulation of HLA-C Expression on Melanocytes May Contribute to the Therapeutic Efficacy of UVB Phototherapy in Psoriasis. Int. J. Mol. Sci..

[10] La Banca V., De Domenico S., Nicolai S., Gatti V., Scalera S., Maugeri M., Mauriello A., Montanaro M., Pahnke J., Candi E. (2024). ABCC1 Is a ΔNp63 Target Gene Overexpressed in Squamous Cell Carcinoma. Int. J. Mol. Sci..

[11] Pacheco-Tovar D., Pacheco-Tovar M.G., Saavedra-Alonso S., Zapata-Benavides P., de-Jesús Torres-Del-Muro F., Bollain-y-Goytia J.J., Herrera-Esparza R., Rodríguez-Padilla C., Avalos-Díaz E. (2024). shRNA-Targeting Caspase-3 Inhibits Cell Detachment Induced by Pemphigus Vulgaris Autoantibodies in HaCaT Cells. Int. J. Mol. Sci..

[12] Bchetnia M., Powell J., McCuaig C., Boucher-Lafleur A.M., Morin C., Dupéré A., Laprise C. (2024). Pathological Mechanisms Involved in Epidermolysis Bullosa Simplex: Current Knowledge and Therapeutic Perspectives. Int. J. Mol. Sci..

